# Au–Nitrogen-Doped Graphene Quantum Dot Composites as “On–Off” Nanosensors for Sensitive Photo-Electrochemical Detection of Caffeic Acid

**DOI:** 10.3390/nano10101972

**Published:** 2020-10-05

**Authors:** Qiyuan Zhao, Lin Zhou, Xue Li, Jiaqi He, Weichun Huang, Yan Cai, Jin Wang, Tingting Chen, Yukou Du, Yong Yao

**Affiliations:** 1 School of Chemistry and Chemical Engineering, Nantong University, Nantong 226019, China; 1908110107@stmail.ntu.edu.cn (Q.Z.); 1908320003@stmail.ntu.edu.cn (L.Z.); 1808031049@stmail.ntu.edu.cn (X.L.); 1808031043@stmail.ntu.edu.cn (J.H.); huangweichun@ntu.edu.cn (W.H.); yancai2010@ntu.edu.cn (Y.C.); 2 College of Chemistry, Chemical Engineering and Materials Science, Soochow University, Suzhou 215123, China

**Keywords:** photo-electrochemical detection, local surface plasmon resonance effect, Au/NGQDs, biological molecules, nanosensor

## Abstract

Herein, gold–nitrogen-doped graphene quantum dot (Au/NGQD) composite modified electrodes were fabricated and applied as “on–off” nanosensors for the photo-electrochemical (PEC) detection of caffeic acid under visible-light irradiation. An effective and simple strategy was established for the preparation of Au/NGQD composites by hydrothermal and calcination methods. Owing to the quantum confinement effect of NGQDs, the local surface plasmon resonance (LSPR) effect of Au nanoparticles (NPs), and the synergistic effect between Au and NGQDs NPs, the Au/NGQDs showed excellent PEC performance, with wide linear concentration ranges (0.11 to 30.25 μM and 30.25 to 280.25 μM), a low detection limit (0.03 μM), excellent sensitivity, and high stability. The present study may provide an advanced strategy for the simple design of Au/NGQD composites to allow their effective application for selective and sensitive sensing of small biological molecules.

## 1. Introduction

Caffeic acid (CA), one of the plant-derived polyphenols, mainly exists in plants and fruits, and plays a vital role in human health due to its unique biological activities. Relevant studies showed that moderate concentrations of CA in cosmetics, food, and drugs were effective in slowing aging, reducing inflammation, and preventing cancer [1]. However, an anomalous content of CA in the human body can irritate the skin and mucous membranes, and can even cause cancer [2]. Hence, it is of great significance to establish a valid method or technique for quantitative detection of CA to achieve a healthy daily diet. Various methods and strategies have been developed for CA detection, such as an electrochemical method [3], fluorometric assay [4], high-performance liquid chromatography [5], supercritical fluid extraction [6], and photo-electrochemical (PEC) detection [7]. Compared with the above methods, PEC has more advantages, including having a wider detection range, lower detection limit, and higher sensitivity. Nevertheless, the photoactive material is a vital factor for the fabrication of photo-electrochemical biosensors.

The great success achieved in the photo-electrochemical field until now is triggering immense enthusiasm for exploring highly photoactive materials. Transition metal dichalcogenides, titanium dioxide, carbon nitride, quantum dots, and noble metals are the most sought after photoactive materials [8,9,10,11,12]. Recently, nitrogen-doped graphene quantum dots (NGQDs), which have unique quantum effects, substantial photostability, and good biocompatibility, have been widely applied in electronics, for photocatalysis, as well as in biosensors [13,14,15]. However, the tiny size of NGQDs leads to their accumulation, which decreases the photochemical response in the PEC reaction process. In addition, their slow electron conductivity also limits the performance of NGQDs in PEC detection [16].

Until now, the effective way to address the aforementioned shortcomings of NGQDs has been established by combining high conductive two-dimensional material with them. Shi constructed NGQD-ZnNb_2_O_6_/g-C_3_N_4_ heterostructures as the photocatalysts for hydrogen production under visible-light irradiation, and observed that the photocurrent was increased rapidly when the light source was turned on. The photocurrent intensity of NGQD-ZnNb_2_O_6_/g-C_3_N_4_ was much higher than for ZnNb_2_O_6_ and g-C_3_N_4_, which was mainly due to the efficient charge separation and enhanced photocatalytic activity of the Zn/7CN catalyst [17]. Zhu prepared gold–nitrogen-doped graphene quantum dot (Au/NGQD) nanoparticles (NPs) and studied their integrated enhancement mechanisms, finding the potential of Au/NGQD NPs to be superior to Surface Enhanced Raman Scattering (SERS) substrates for on-site Raman detection, owing to their electromagnetic and chemical enhancement [16]. You developed a sensitive electrochemiluminescence (ECL) aptasensor for zearalenone detection based on NH_2_-Ru@SiO_2_ NPs, showing a wide linear range of 10 fg mL^−1^ to 10 ng mL^−1^ and a low detection limit of 1 fg mL^−1^. Gold nanoparticles (Au NPs) have the advantages of being chemically inert, oxidation-free, and highly biocompatible, which is important in biosensing applications [18]. Moreover, various synthetic strategies for preparing AuNPs of different shapes and sizes have been explored, resulting in the ability to adjust their plasmonic functionalities, as well as allowing their application in the fields of biotherapy, catalysis, and sensing [19,20,21,22]. Many reports showed that the photocurrent response in NGQD/metallic NPs was higher than bare metallic NPs, which is mainly due to the charge transfer between the detection molecules and NGQD/metallic NPs can enhance the response signals, and π-π staking between the NGQDs and absorbed molecules can shorten the distance between the molecules and the substrate which will further enhance the detection signals. Moreover, when compared to conventional graphene sheets, nitrogen-doped graphene quantum dots (NGQDs) have larger specific surface areas and more accessible edges, which lead to more effective adsorption of target molecules [23,24]. Therefore, a photo-electrochemical sensing platform of Au nanoparticles (NPs) and NGQDs with making the most use of their quantum effects and local surface plasmon resonance (LSPR) effects would give promising and interesting analysis results.

In this work, an effective and simple strategy was established for the preparation of Au/NGQD composites by hydrothermal and calcination methods, which could be employed as “on–off” sensing platforms for sensitive photo-electrochemical detection of caffeic acid under visible-light irradiation. Herein, the experimental conditions that might influence the CA detect were studied and optimized. The possible reaction mechanism towards the PEC process of Au/NGQD composites was also discussed and is presented. Specifically, under visible-light irradiation, NGQDs absorb light energy and generate NGQD* free radicals for CA oxidation. Meanwhile the Au NPs are excited and generate a local surface plasmon (LSPR) effect. The Au NPs recognize the CA quickly and transmit electrons in the sensing system [7]. Thus, the synergistic effect between Au nanoparticles and NGQDs can accelerate the PEC process effectively, resulting in sensitive and stable photocurrent performance. As a result, the linear concentration ranges of CA were calculated to be between 0.11 and 30.25 μM and between 30.25 and 280.25 μM, while the detection limit was about 0.03 μM, as determined using the amperometric i-t technique.

## 2. Materials and Methods

### 2.1. Materials and Apparatus

The materials and apparatus used in this work are supplied in the Supplementary Materials.

### 2.2. Preparation of Au, NGQDs, and Au/NGQD Composites

Au nanoparticles were prepared using the following steps. Firstly, 1.8 mL 0.1 M citric acid and 4.2 mL 0.1 M trisodium citrate were added into secondary distilled water consecutively while it was boiling. Next, the solution was stirred for 15 min in open air conditions to partially oxidize the citrate. Secondly, 25.4 mM HAuCl_4_ was injected into the above-mentioned solution and stirred for another 3 min, then the mixture solution was transferred into ice water. When the solution turned bright red, this indicated that the Au colloid had been successfully prepared. The NGQDs were fabricated using the calcination method reported in our previous work [7]. In detail, 3.37 g citric acid and 2.86 g urea were added into a crucible and maintained at 200 °C for 8 h. Ammonia gas and water vapor were released during the calcination process. The obtained black powder was dispersed into water without further ultrasonic treatment.

The Au/NGQDs (mAu: mNGQDs = 1.1:20) nanocomposites were constructed using the following step: typically, 10 mL Au (0.056 mM/L, 0.11 mg/L) and 10 mL NGQDs (2 mg/mL) were mixed, then the mixture was treated under ultrasonication for 2 h to ensure they were mixed thoroughly.

### 2.3. Fabrication of Au, NGQD, and Au/NGQD Composite Modified Electrodes

The Au, NGQDs, and Au/NGQDs composite modified electrodes were fabricated by dropping a dispersion containing 10 μL of Au, NGQDs, and Au/NGQDs composites onto the surface of a polished glassy carbon electrode (GCE). The distances between modified electrodes and visible light source were set to 15 cm, which was confirmed as the optimum condition in our previous work [25].

## 3. Results and Discussion

### 3.1. Detection Strategy of Au/NGQD Composites for CA

**Scheme 1:**
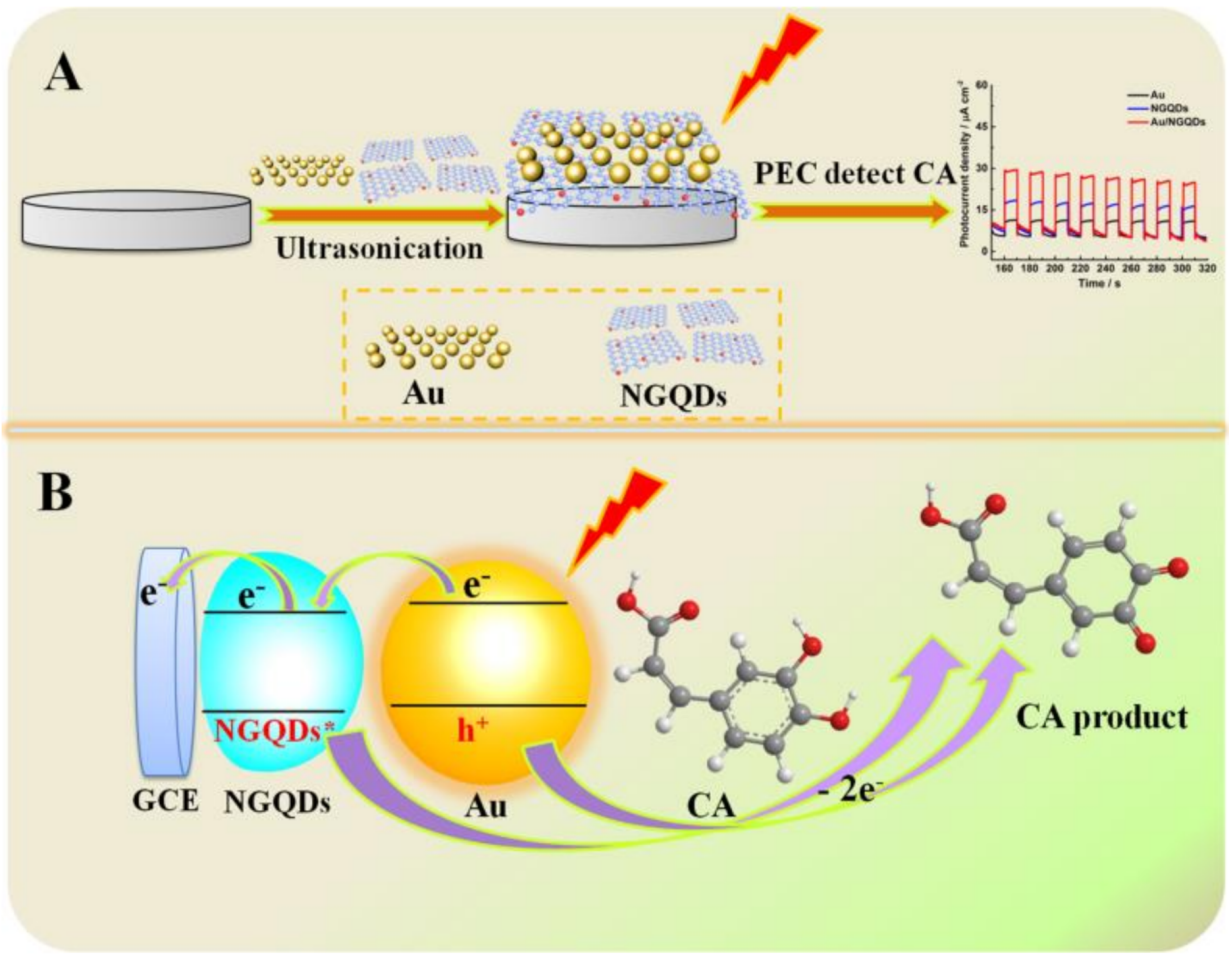
(**A**) Construction process of the proposed gold–nitrogen-doped graphene quantum dot (Au/NGQDs) sensing platform for the photo-electrochemical (PEC) detection of caffeic acid (CA). (**B**) Schematic illustration of the sensing process.

A sensitive “Au NPs” sensing platform based on Au/NGQD composites for photo-electrochemical (PEC) detection of caffeic acid (CA) was fabricated and applied in this work. The detection strategy was based on the oxidation of CA by Au and NGQDs due to the LSPR and quantum effects under visible-light irradiation. As depicted in Scheme 1A, when the light was turned on, the Au nanoparticles were effectively lit up, generating the local surface plasmon effect, leading to the Au NPs being further excited to produce the photo-generated electron holes. The photogenerated holes recognize and oxidize CA rapidly, meanwhile the electrons would transfer to the electrode system. Meanwhile, the NGQDs on the GCE surface are excited, allowing them to oxidize the hydroxyl groups (-OH) in the CA molecules. Then, the NGQDs are reduced to NGQDs^•−^ in the PEC redox process [26,27]. As a result, the synergistic effect between Au nanoparticles and NGQDs promotes the detection process and produces a high photocurrent response. Details of PEC sensing process are presented in Scheme 1B. The above strategies provide excellent results in terms of PEC detection of CA using the Au/NGQDs sensing platform, including having high sensitivity, a wide detect concentration range, as well as good stability.

### 3.2. Characterization of Au, NGQDs, and Au/NGQDs Composite Modified Electrodes

**Figure 1:**
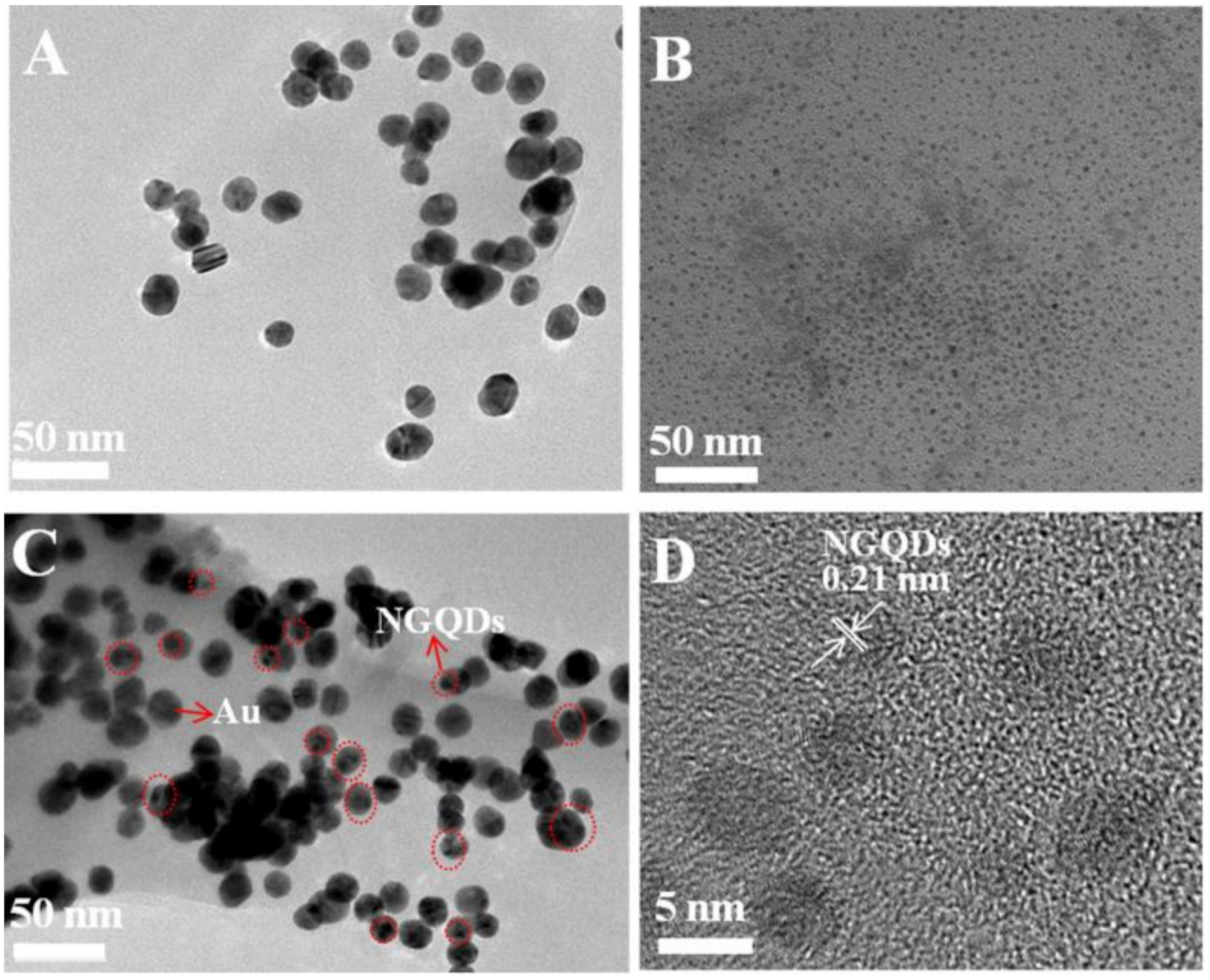
TEM images of (**A**) Au, (**B**) nitrogen-doped graphene quantum dots (NGQDs), and (**C**) Au/NGQDs. HRTEM image of (**D**) NGQDs.

The Au, NGQDs, and Au/NGQD composites were characterized to investigate their morphology, size, crystallinity, and the ability to cause a local surface plasmon effect. Figure 1A,B demonstrate that the spherical Au and NGQD NPs were dispersed uniformly on the surfaces of electrodes, with size ranges of about 18 ± 3.8 nm and 5.1 ± 0.6 nm. The High-Resolution Transmission Electron Microscopy (HRTEM) image of NGQDs in Figure 1D shows the single crystalline structure with a lattice fringe of 0.21 nm, which was attributed to the typical (1120) lattice plane of graphene. Figure 1C shows that the NGQD NPs were dispersed on the surfaces of Au NPs. XRD patterns of Au/NGQDs in Figure S1A show the typical diffraction peak of NGQD located at 27.77°, corresponding to the (002) plane of graphene. The peaks that appeared at 38.34°, 44.27°, 64.12°, and 77.69° were mainly attributed to the (111), (200), (220), and (311) crystal facets of the Au NPs. These were slightly shifted compared with the standard patterns for Au (JCPDS, 04-0784), which was attributed to the formation of Au and NGQDs composites under ultrasonication. In Figure S1B, pure Au NPs showed a typical absorption edge at 522 nm, which was mainly due to the strong localized surface plasmon resonance (LSPR) bands in Au NPs. The NGQDs presented two UV-vis absorption edges at 340 and 408 nm, which were mainly ascribed to the π→π* transition of the conjugated graphene network structure, while the other strong band at 400 nm was assigned to the n-π* electronic transitions linked with the nitrogen and oxygen free electron pairs. Figure S1B reveals the typical UV-Vis absorption bands for Au and NGQDs. There was almost no shift in comparison with pure Au and NGQDs, demonstrating the successful preparation of Au/NGQD composites.

**Figure 2:**
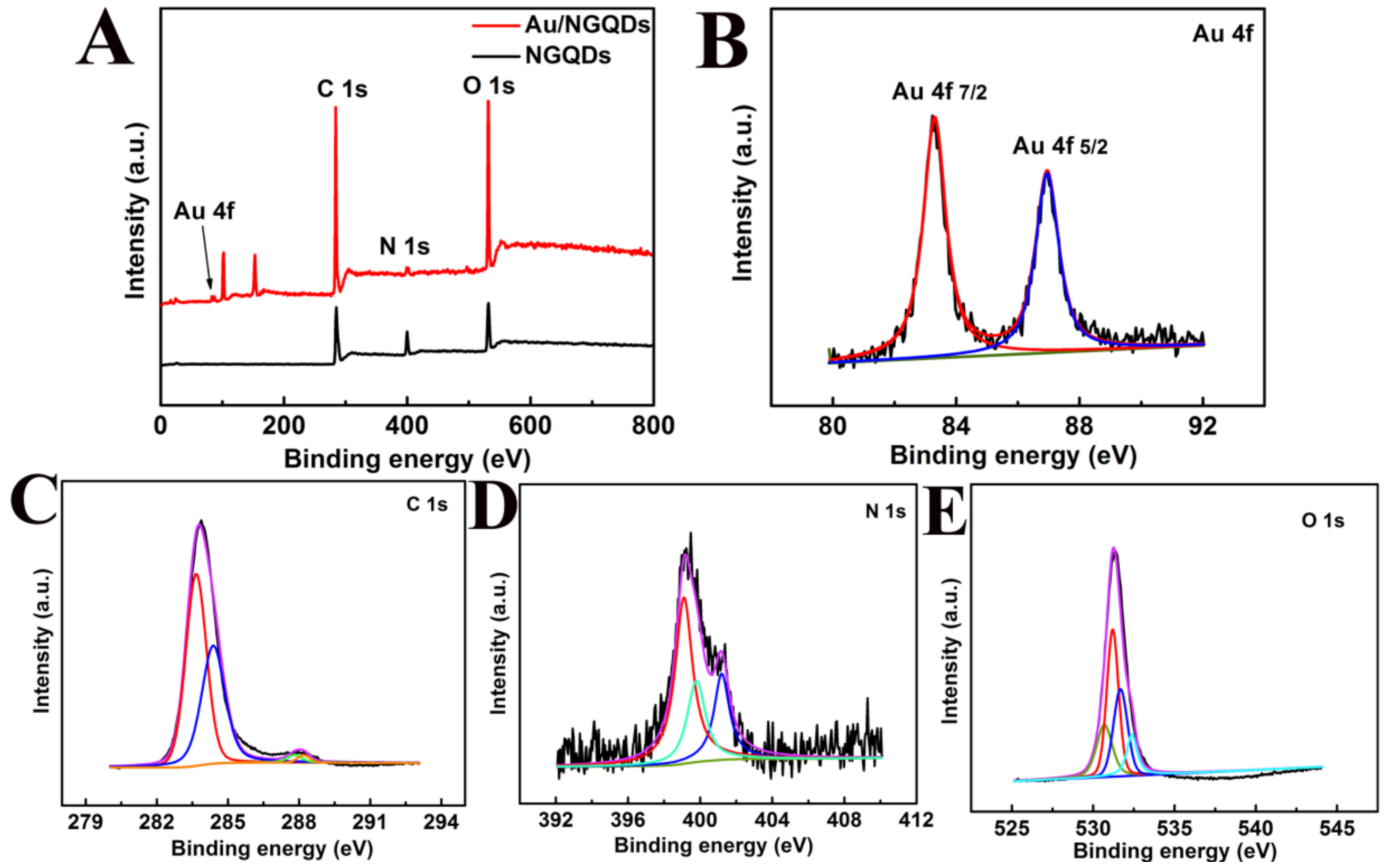
(**A**) XPS full scan spectra of NGQDs and gold nanoparticle (AuNP)/NGQDs. (**B**–**E**) The XPS analyses of Au 4f, C 1s, N 1s, and O 1s of AuNP/NGQDs, respectively.

To further demonstrate the successful synthesis of AuNP/NGQDs, the chemical compositions of NGQDs and AuNP/NGQDs were investigated by X-ray photoelectron spectroscopy (XPS), as shown in Figure 2. The XPS full-scan spectrum of NGQDs displayed three main peaks of C 1s, N1s, and O 1s at 284.88, 400.08, and 533.08 eV, respectively; while that of AuNP/NGQDs presented three main peaks of C 1s, N 1s, and O 1s at 286.08, 399.08, and 534.08 eV, and a minor peak of Au 4f at 84.38 eV. The high-resolution C 1s XPS spectrum was divided into four peaks, including C=C at 283.59 eV, C-N at 284.19 eV, C-O at 287.79 eV, and O-C=O at 288.19 eV, indicating the abundant carbon structure. The N 1s XPS spectrum comprised three peaks with binding energies of 398.79, 399.09, and 401.19 eV, corresponding to pyridine, pyrrolic, and graphitic nitrogen, respectively. Furthermore, the peak of O 1s was fitted into four peaks of 530.69, 531.20, 531.68, and 532.38 eV corresponding to C-OH, C=O, C-O-C, and C-O, respectively, indicating the existence of numerous oxygenated functional groups [28]. Moreover, the peaks located at 102.79 and 150.49 eV corresponded to the XPS were correspondent to the XPS spectra of Si element. Importantly, the observation of Au 4f in the XPS spectrum of AuNP/NGQDs provided strong evidence of the existence of the AuNP/NGQD nanocomposites.The fitted Au 4f gave binding energy values of 83.29 (Au 4f 7/2) and 86.95 eV (Au 4f 5/2). These were slightly higher binding energy values compared with the XPS spectrum of NGQDs, which might have been caused by an asymmetric structure from Au being adsorbed on NGQDs [29].

### 3.3. Electrochemical andPhoto-Electrochemical Characterization of the Sensing Platform

**Figure 3:**
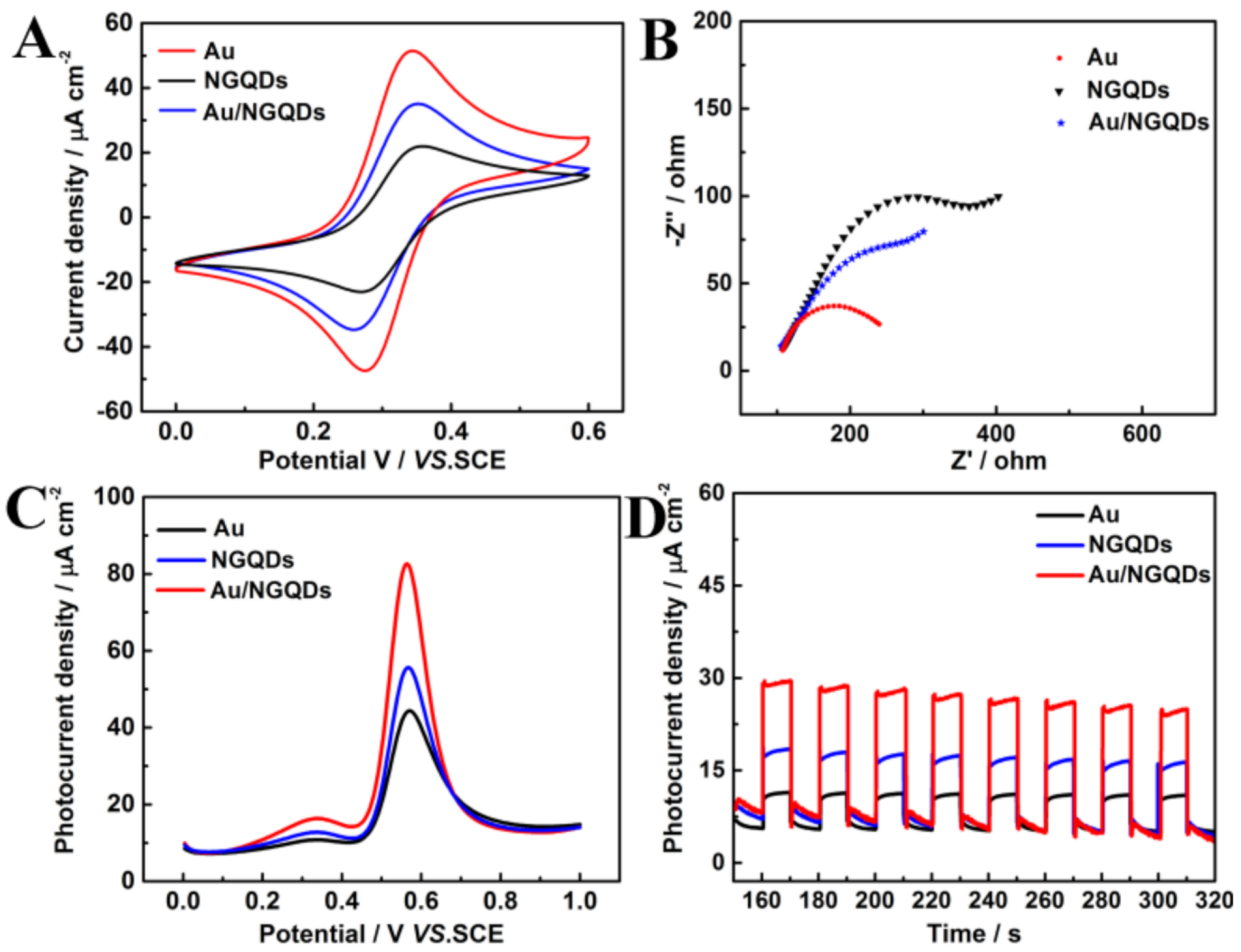
(**A**) The Cyclic voltammetry curve (CVs) and (**B**) Impedance measurement (EIS) spectra of Au, NGQD, and Au/NGQDs electrodes in 1.0 mM K_3_[Fe(CN)_6_]/K_4_[Fe(CN)_6_] containing 0.1 M KCl. (**C**) Differential pulse voltammetry (DPVs) and (**D**) Time-current (i-t) responses of Au, NGQDs, and Au/NGQDs electrodes in 0.5 mM CA containing 0.1 M BR buffer solution (pH = 3.0) under visible-light irradiation. The illumination from a Xe lamp was interrupted every 10 s.

The effective charge transfer is the foremost electrochemical performance indicator for a modified electrode. Herein, the charge transfer abilities of Au, NGQDs, and Au/NGQDs sensing platforms were explored via CVs and EIS techniques in a 2.5 mM K_3_[Fe(CN)_6_]/K_4_[Fe(CN)_6_] solution containing 0.1 M KCl. As depicted in Figure 3A, the NGQDs and Au electrodes showed current densities at about 20.27 and 34.06 μA cm^−2^, respectively, indicating that Au allows faster electron transfer from the redox probe to the working electrode. While modified with Au nanoparticles, the Au/NGQDs electrode demonstrated the highest photocurrent density of about 52.98 μA cm^−2^ when compared with the pure Au and NGQDs electrodes, illustrating that Au nanoparticle could accelerate the electron transfer of semiconductor NGQDs efficiently. Additionally, the CV results were well-matched with the EIS data in Figure 3B, in which the Au and NGQDs electrodes displayed the smallest and largest semicircle diameters, respectively, of the three modified electrodes.

The PEC performances of Au, NGQDs and Au/NGQD composite modified electrodes were investigated by DPV and i-t records in 0.5 mM CA containing 0.1 M BR buffer solution (pH = 3.0) under visible light irradiation. As illustrated in Figure 3C, there was a distinct oxidation peak current for all three modified electrodes at 0.58 V, which was due to the oxidization of the hydroxyl group (-OH) of CA. An oxidation peak current at 44.38 μA cm^−2^ was displayed for the Au electrode, which was attributed to the local surface plasmon effect (LSPR) of Au nanoparticles under visible-light irradiation. The NGQD electrode showed an oxidation peak current at about 55.64 μA cm^−2^, owing to the fact that the NGQDs can absorb light and generate NGQD* free radicals in order to oxidize CA with the assistance of visible-light irradiation. In Figure 3C, the Au/NGQD composite electrode showed the highest oxidation peak current approaching 82.61 μA cm^−2^, compared with Au and NGQDs electrodes, which could be ascribe to the synergistic effect of Au and NGQDs.

**Scheme 2:**
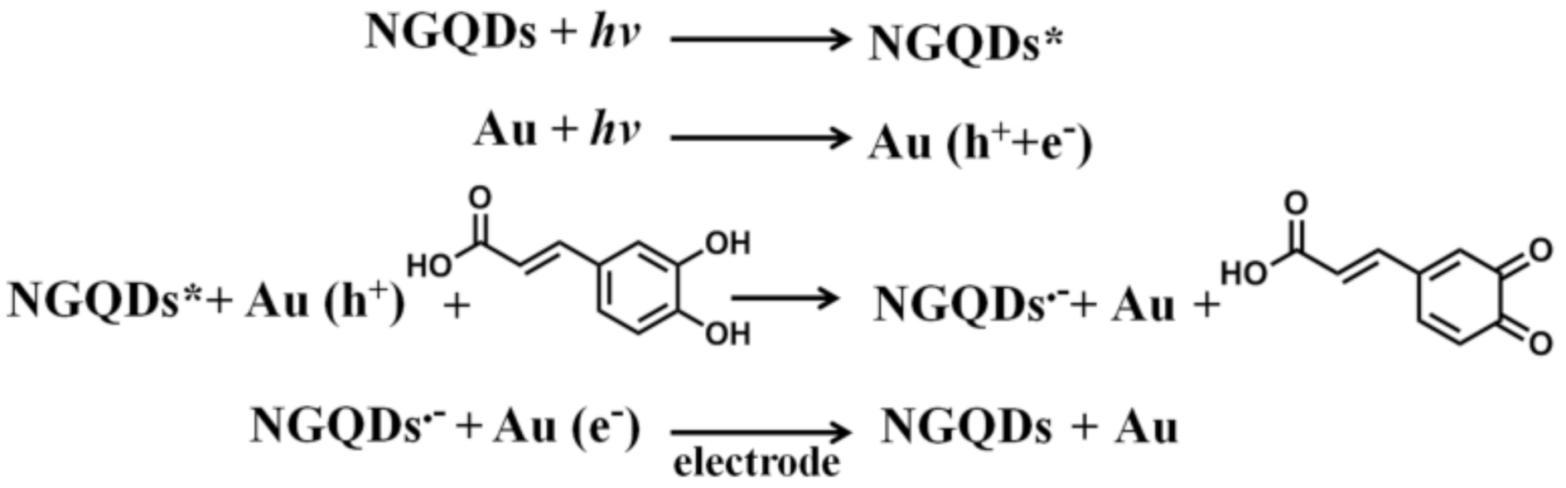
Schematic illustration of the CA reaction process on Au/NGQD/glassy carbon electrodes (GCEs) under visible-light irradiation.

It is noted that the Au/NGQDs sensing platform showed interrupted photocurrent signals in Figure 3D when the light source was turned on and off every 10 s. The photocurrent increased promptly when the light source was turned on, illustrating that the Au NPs and NGQDs could recognize and oxidize the CA immediately under visible light irradiation. The photocurrent decreased quickly when the light source was turned off, which was due to dark conditions that would inhibit the quantum confinement and LSPR effects of the Au/NGQD electrode, thus further inhibiting the oxidation of caffeic acid (Scheme 2). The results in Figure 3 conclusively demonstrate that the Au/NGQD composite sensing platform could be used for PEC detection of CA under visible-light irradiation.

### 3.4. Optimization of Experimental Conditions

**Figure 4:**
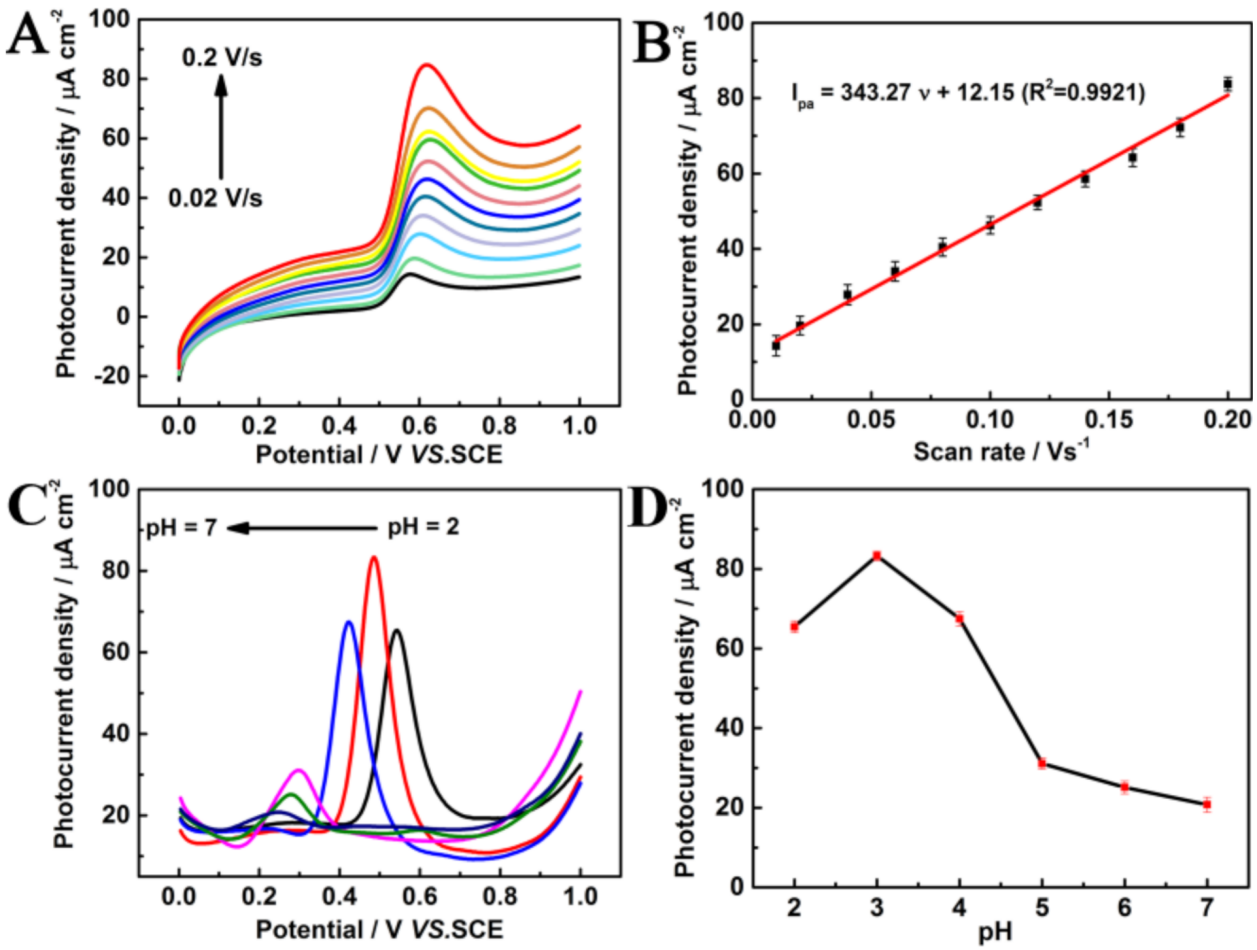
(**A**) Linear sweep voltammetry (LSVs) of Au/NGQD electrodes in 0.1 M BR buffer solution (pH = 3.0) containing 0.5 mM CA in the scan rates ranging from 0.2 to 0.02 V s^−1^ under visible-light illumination. (**B**) The calibration curves of photocurrent density responses versus the scan rates. (**C**) CVs of the Au/NGQD electrodes in 0.1 mM CA, with pH values ranging from 2.0 to 7.0. (**D**) The plots of the anodic peak potential against the pH.

The pH and scan rate are crucial factors in PEC performance. In Figure 4A, the effect of scan rate during the CA detection process was studied by linear sweep voltammetry (LSV) records in 0.5 mM CA under visible-light illumination. As illustrated in Figure 4A, the oxidation current density increased rapidly, with scan rates ranging from 0.02 to 0.2 V/s. The linear regression equation between the oxidation peak current density and scan rate was I_pa_ = 343.27*v* + 12.15 (R^2^ = 0.9921), demonstrating the electron transfer between the Au/NGQD electrodes and CA was the surface-adsorption-controlled process. The photocurrent responses of Au/NGQD electrodes in different pH (ranging from 2.0 to 7.0) solutions was studied and is shown in Figure 4C. As can be seen, the highest oxidation peak current was obtained in pH = 3.0 solution. The oxidation peak current decreased sharply with the increase of the pH value, illustrating that pH = 3.0 is the optimal condition for the oxidation of CA.

### 3.5. Analytical Performance

**Figure 5:**
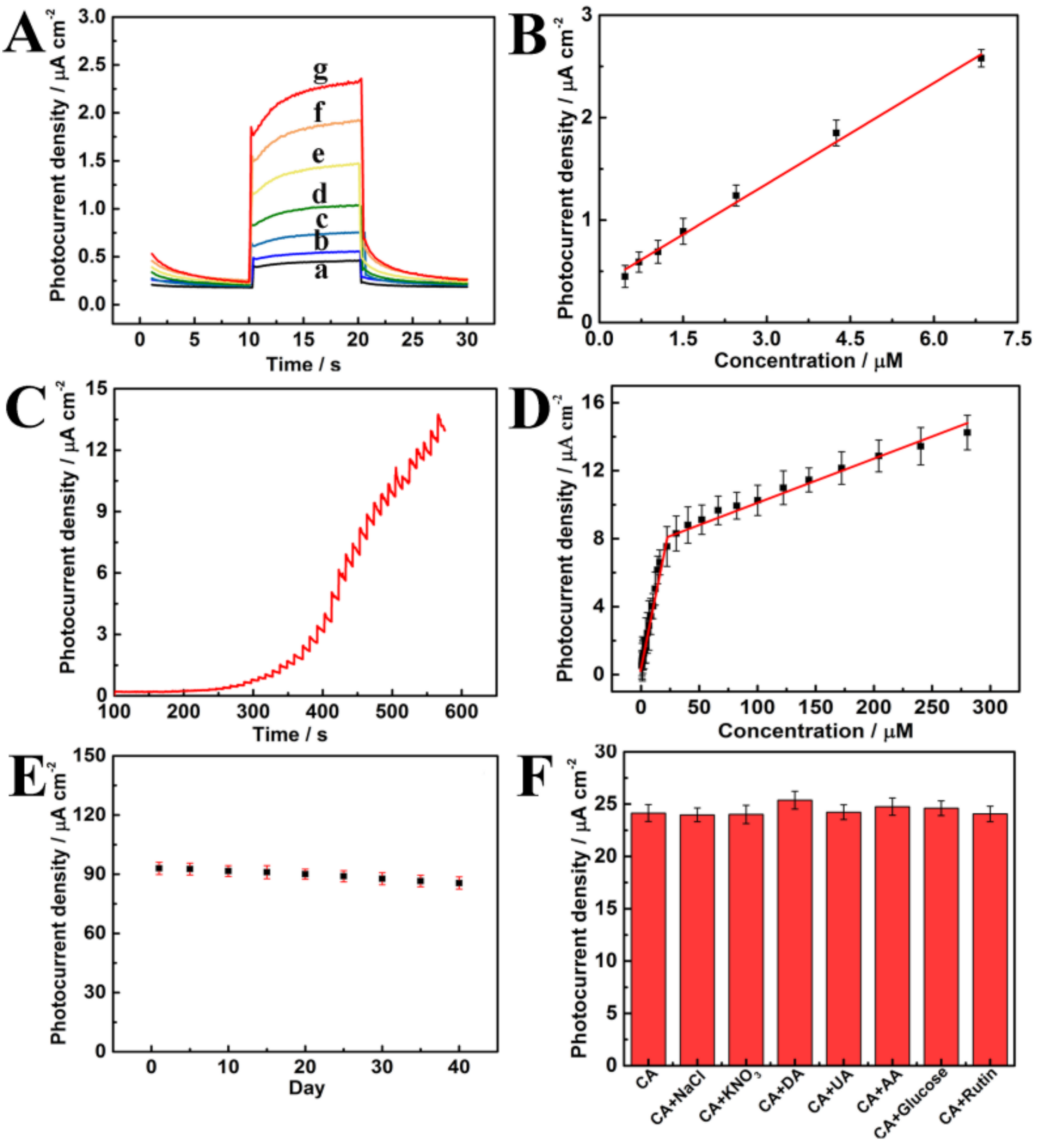
(**A**) The i-t performance of Au/NGQD electrodes in different concentrations of CA: (**a**–**g**) 0.46, 0.71, 1.05, 1.50, 2.45, 4.25, and 6.85 μM, respectively. (**B**) Plot of photocurrent density vs. CA concentration. (**C**) Transient photocurrent density vs. time plotted for the Au/NGQD electrodes in BR buffer solutions (pH 3.0) containing different concentrations of CA, ranging from 0.11 to 30.25 μM and from 30.25 to 280.25 μM. (**D**) Plots of photocurrent density vs. the concentration of CA. (**E**) The stability of Au/NGQD electrodes. (**F**) The anti-interference effect of Au/NGQD electrodes for CA detection and different interfering substance solutions.

To investigate the analytical performance of the sensing platform, the photocurrent response of the developed Au/NGQD electrodes was employed in Britton-Robinson (BR) buffer solutions (pH 3.0) containing various concentrations of CA under the optimal testing conditions (Figure 5). As can be observed in Figure 5B, the photocurrent density increased linearly with the increase of the trace CA solution. The linear regression equation was I = 343.27*c* + 12.15 (R^2^ = 0.9921). Furthermore, the PEC response of Au/NGQD electrodes was also examined by i-t curves with various CA solutions. As displayed in Figure 5C, the photocurrent densities increased with increasing CA concentration from 0.11 to 30.25 μM and from 30.25 to 280.25 μM under visible-light illumination. The linear regression equation was I = 0.38*c* + 0.30 (R^2^ = 0.9951), I = 0.023*c* + 8.01 (R^2^ = 0.9937), with a detection limit of 0.03 μM. Table S1 is the comparison results between this work and other methods for CA detection. It could be observed that Au/NGQD electrodes had a wide linear concentration range and low detection limit, indicating the successful fabrication of Au/NGQD nanosensors for CA detection.

To verify the stability of the fabricated PEC sensing platform, the Au/NGQD electrodes were stored at room temperature for 40 days. The results in Figure 5E show that the photocurrent density of the PEC sensing platform remained at 92.05% of its initial state.

To evaluate the selectivity and anti-interference effect of Au/NGQDs electrodes for caffeic acid detection, dopamine (DA), uric acid (UA), ascorbic acid (AA), glucose (Glu), and rutin (Ru) with similar structures were selected as potential interferants. In Figure 5F, there was no significant change in the photocurrent density on Au/NGQD electrodes when the CA was mixed with different interfering substances, except with the addition of DA solution. Three reasons may account for the above phenomenon. Firstly, the excellent photocurrent response of Au/NGQD electrodes in the CA solution was attribute to the formation of the stable conjugated π bonds in the CA structure, which meant that the oxhydryl groups in the CA molecules were easily oxidized. Secondly, the photocurrent density response of Au/NGQD electrodes in the DA–CA mixed solution showed an obvious increase due to the oxidation of oxhydryl groups in DA molecules. Thirdly, though the oxhydryl or carbonyl group existed in these interfering substances (UA, AA, Glu, Ru), unstable oxidation products or large molecular weights would make their oxidization difficult.

## 4. Conclusions

As mentioned above, a sensitive and simple Au/NGQDs composite was developed and applied in the photo-electrochemical detection of caffeic acid. The fabricated Au/NGQDs photo-electrochemical sensor showed an outstanding PEC response, with a low detection limit (0.03 μM) and wide detection range (0.11 to 30.25 μM and 30.25 to 280.25 μM). The great photo electrochemical response signals on Au/NGQD sensing platform was due to the local surface plasmon effect of Au NPs, the quantum effects of NGQDs and the synergistic effect of Au and NGQDs. The prepared Au/NGQDs composite sensing platform opens a new avenue for highly reliable small biomolecules detections and show a bright future for photoelectrochemical bioanalysis.

## Supplementary Materials

The following are available online at https://www.mdpi.com/2079-4991/10/10/1972/s1, Figure S1.: (A) XRD patterns of Au/NGQDs, (B) UV-visible spectra of Au, NGQDs and Au/NGQDs, Table S1.: Comparison of this work with other methods for CA detection.
