# Supplementary material for: Au–Nitrogen-Doped Graphene Quantum Dot Composites as “On–Off” Nanosensors for Sensitive Photo-Electrochemical Detection of Caffeic Acid
Source: Nanomaterials (Basel). 2020 Oct 5;10(10):1972. doi: 10.3390/nano10101972 (PMC7599707; doi:10.3390/nano10101972)
Supplement: Supplementary file 1 [file nanomaterials-10-01972-s001.pdf]

nanomaterials

Supplementary Materials

Materials and Apparatus

Caffeic acid, dopamine, ascorbic acid, uric acid, rutin, glucose, NaCl, KNO_3 urea, citric acid, and trisodium citrate were obtained from Sinopharm Chemicals Reagent Co., Ltd. (Shanghai, China). The Britton–Robinson (BR) buffer solutions were prepared with phosphoric acid, boric acid, and glacial acetic acid aqueous solutions and used as supporting electrolytes. The pH values of the electrolyte solutions in the experiments were adjusted with NaOH solution. Double-distilled water was used throughout the experiment. All chemicals were of analytical grade and used without any further purification.

Scanning electron microscopy (SEM) images recorded on a S-4700 system (Hitachi High Technologies Corporation, Tokyo, Japan) and high-magnification transmission electron microscopy (TEM) using an FEI TecnaiF20 TEM (FEI Company, Portland, OR, USA) at an accelerating voltage of 200 kV were employed to characterize the morphologies of Au NPs and NGQDs. The chemical compositions and structures of Au NPs and NGQDs were obtained by X-ray diffraction (XRD; PANalytical X'Pert PRO MRD XRD; CuKα radiation; λ = 1.54056 Å; PANalytical Co., Almelo, Holland). An electrochemical workstation (CHI 760e; Shanghai Chen Hua Instrumental Co., Ltd., Shanghai, China) equipped with a standard three-electrode system was employed to conduct all electrochemical and photoelectrochemical measurements, in which the glassy carbon electrode (GCE; diameter: 3.0 mm), platinum electrode, and Ag-AgCl electrode acted as the working electrode, reference electrode, and counter electrode, respectively. A xenon lamp (150 W) equipped with a UV cut-off filter (>400 nm) was used as the visible light source.

Preparation of Au, NGQDs, and Au/NGQD Composites

Au nanoparticles were prepared using the following steps. Firstly, 1.8 mL 0.1 M citric acid and 4.2 mL 0.1 M trisodium citrate were added into secondary distilled water consecutively while it was boiling. Then, the solution was stirred for 15 min in open air conditions to partially oxidize the citrate. Secondly, 25.4 mM $HAuCl_4$ was injected into the above-mentioned solution and stirred for another 3 min. Finally, the mixture solution was transferred into ice water. When the solution turned bright red, the Au colloid was successfully prepared. The NGQDs were fabricated using the calcination method reported in our previous work. The Au/NGQD composites were prepared by ultrasonication a certain volume of a dispersion of Au and NGQDs.

Fabrication of Au, NGQD, and Au/NGQD Composite Modified Electrodes

The Au, NGQD, and Au/NGQD composite modified electrodes were fabricated by dropping 10 μL of Au, NGQD, and Au/NGQD composite dispersions onto the surface of the polished glassy carbon electrodes (GCEs). The distance between the modified electrodes and the visible light source was set as 15 cm, which was confirmed as the optimum condition in our previous report [1].

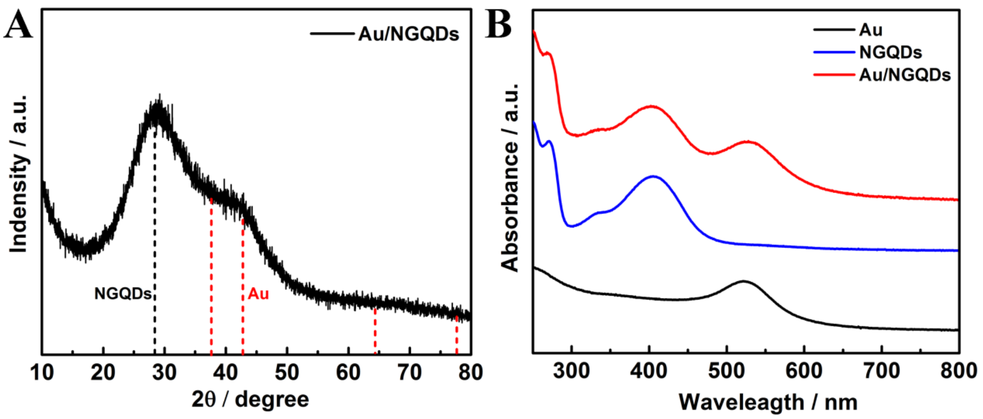

Figure S1. (**A**) XRD patterns of Au/NGQDs. (**B**) UV-visible spectra of Au, NGQDs, and Au/NGQDs.

Table S1. Comparison of this work with other methods for CA detection.

Methods	Linear range (μM)	Detection limit (μM)	Ref.
Electrochemistry	0.74–10.5	0.15	2
Fluorometry	2–350	0.2	3
Fluorometry	0.14–1.4	0.06	4
Square-wave stripping voltammetry	0.1–10	0.091	5
HPLC	2–555	2	6
Photo-electrochemistry	0.11–280.25	0.03	This work

References

1. Wang, J.; Zhang, K.; Xu, H.; Yan, B.; Gao, F.; Shi, Y.; Du, Y. Engineered photoelectrochemical platform for the ultrasensitive detection of caffeic acid based on flower-like MoS2 and PANI nanotubes nanohybrid. *Sensors Actuators B: Chem.* **2018**, *276*, 322–330, doi:10.1016/j.snb.2018.08.128.
2. Diaconu, M.; Litescu, S.-C.; Radu, G.-L. Laccase–MWCNT–chitosan biosensor—A new tool for total polyphenolic content evaluation from in vitro cultivated plants. *Sensors Actuators B: Chem.* **2010**, *145*, 800–806, doi:10.1016/j.snb.2010.01.064.
3. Cai, N.; Li, Y.; Chen, S.; Su, X. A fluorometric assay platform for caffeic acid detection based on the G-quadruplex/hemin DNAzyme. *Anal.* **2016**, *141*, 4456–4462, doi:10.1039/C6AN00543H.
4. Xiang, X.; Shi, J.; Huang, F.; Zheng, M.; Deng, Q. Quantum dots-based label-free fluorescence sensor for sensitive and non-enzymatic detection of caffeic acid. *Talanta* **2015**, *141*, 182–187, doi:10.1016/j.talanta.2015.04.002.
5. Filik, H.; Çetintaş, G.; Avan, A.A.; Aydar, S.; Koç, S.N.; Boz, I. Square-wave stripping voltammetric determination of caffeic acid on electrochemically reduced graphene oxide–Nafion composite film. *Talanta* **2013**, *116*, 245–250, doi:10.1016/j.talanta.2013.05.031.
6. Wang, H.; Provan, G.J.; Helliwell, K. Determination of rosmarinic acid and caffeic acid in aromatic herbs by HPLC. *Food Chem.* **2004**, *87*, 307–311, doi:10.1016/j.foodchem.2003.12.029.

© 2020 by the authors. Licensee MDPI, Basel, Switzerland. This article is an open access article distributed under the terms and conditions of the Creative Commons Attribution (CC BY) license (http://creativecommons.org/licenses/by/4.0/).
